# Alcohol Consumption and Binge Drinking During Pregnancy Among Adults Aged 18–49 Years — United States, 2018–2020

**DOI:** 10.15585/mmwr.mm7101a2

**Published:** 2022-01-07

**Authors:** Lucas K. Gosdin, Nicholas P. Deputy, Shin Y. Kim, Elizabeth P. Dang, Clark H. Denny

**Affiliations:** ^1^Division of Birth Defects and Infant Disorders, National Center on Birth Defects and Developmental Disabilities, CDC; ^2^Epidemic Intelligence Service, CDC.

There is no known safe amount of alcohol consumption during pregnancy; drinking alcohol during pregnancy can cause fetal alcohol spectrum disorders and might increase the risk for miscarriage and stillbirth (*1*). The prevalence of drinking among pregnant women increased slightly during 2011–2018; however, more recent estimates are not yet reported (*2*). CDC estimated the prevalence of self-reported current drinking (at least one alcoholic drink in the past 30 days) and binge drinking (consuming four or more drinks on at least one occasion in the past 30 days) among pregnant adults aged 18–49 years, overall and by selected characteristics, using 2018–2020 Behavioral Risk Factor Surveillance System (BRFSS) data. During 2018–2020, 13.5% of pregnant adults reported current drinking and 5.2% reported binge drinking: both measures were 2 percentage points higher than during 2015–2017. Pregnant adults with frequent mental distress were 2.3 and 3.4 times as likely to report current and binge drinking, respectively, compared with those without frequent mental distress. In addition, pregnant adults without a usual health care provider were 1.7 times as likely to report current drinking as were those with a current provider. Alcohol consumption during pregnancy continues to be a serious problem. Integration of mental health services into clinical care and improving access to care might help address alcohol consumption and mental distress during pregnancy to prevent associated adverse outcomes (*3*).

BRFSS is an annual, state-based, random-digit-dialed telephone survey of health-related behaviors representative of noninstitutionalized adults aged ≥18 years in the United States and participating territories. CDC analyzed 2018–2020 BRFSS self-reported data from 6,327 pregnant adults aged 18–49 years from all 50 U.S. states* and the District of Columbia. The analysis included all pregnant respondents irrespective of gender identity. In 2018, 2019, and 2020, median BRFSS response rates were 49.9% (range = 38.8%–67.2%), 49.4% (37.3%–73.1%), and 47.6% (34.5%–67.2%), respectively.^†^

Persons who reported their sex at birth as female were asked if they were currently pregnant. Current drinking^§^ and binge drinking^¶^ were defined based on 2020–2025 Dietary Guidelines for Americans.** Sociodemographic and health characteristics examined in this analysis included age, race/ethnicity, education, employment status, marital status, having a usual health care provider,^††^ and experiencing frequent mental distress.^§§^

Among pregnant adults, CDC estimated the prevalence and 95% CIs for current and binge drinking, overall and by sociodemographic and health characteristics and U.S. Department of Health and Human Services (HHS) region.^¶¶^ Multivariable regression was used to estimate adjusted prevalence ratios (aPRs) and 95% CIs to identify factors associated with current and binge drinking. To understand potential differences associated with the COVID-19 pandemic, the overall prevalence estimates were examined by year. Differences by year and HHS region were examined using Rao-Scott chi-square tests. Data were weighted to represent state-level population estimates and aggregated to represent regional and national estimates. P-values <0.05 and 95% CIs of aPRs that excluded 1.0 were considered statistically significant. SAS statistical software (version 9.4; SAS Institute) SURVEY procedures were used to account for complex sampling. This activity was reviewed by CDC and was conducted consistent with applicable federal law and CDC policy.***

Among pregnant adults, 13.5% reported current drinking and 5.2% reported binge drinking (38.5% of current drinkers) (Table). The prevalence of current drinking did not differ significantly by year: 11.8% (95% CI = 9.6–14.1) in 2018, 14.6% (11.2%–17.9%) in 2019, and 14.3% (10.5%–18.1%) in 2020 (p = 0.40). The prevalence of binge drinking also did not differ significantly by year: 3.8% (2.4%–5.2%) in 2018, 5.8% (3.2%–8.4%) in 2019, and 6.1% (2.4%–9.7%) in 2020 (p = 0.38). Current drinking differed by age, education, employment, and marital status, and binge drinking differed by employment and marital status. Pregnant adults reporting frequent mental distress had approximately twice the prevalence of current drinking (aPR = 2.3 [1.7–3.1]) and approximately three times the prevalence of binge drinking (aPR = 3.4 [1.9–5.8]) as did those not reporting frequent mental distress. Pregnant adults without a usual health care provider more frequently reported current drinking (17.8%; aPR = 1.7 [1.2–2.3]) than did those with a usual provider (11.9%). Current drinking varied somewhat by HHS Regions (Figure), although differences were not statistically significant (p = 0.25).^†††^

**TABLE:** Estimated prevalence* and adjusted prevalence ratios of current drinking^†^ and binge drinking^§^ reported by pregnant adults aged 18–49 years (N = 6,327), by selected characteristics — Behavioral Risk Factor Surveillance System, United States, 2018–2020

Characteristic	Current drinking		Binge drinking	
	% (95% CI)	aPR^¶^ (95% CI)	% (95% CI)	aPR^¶^ (95% CI)
**Overall**	**13.5 (11.7–15.4)**	**—**	**5.2 (3.6–6.7)**	**—**
**Age group, yrs**				
18–24	16.8 (12.3–21.4)	1.0 (0.7–1.3)	8.5 (4.6–12.4)**	1.4 (0.8–2.6)
25–29	10.3 (7.5–13.1)	0.6 (0.5–0.8)	NA^††^	0.6 (0.3–1.2)
30–34	11.1 (7.5–14.6)	0.6 (0.4–0.9)	NA^††^	1.1 (0.5–2.3)
35–49	17.0 (13.8–20.2)	Ref	4.4 (2.8–6.1)	Ref
**Race/Ethnicity**				
White, non-Hispanic	12.7 (10.9–14.5)	1.1 (0.7–1.6)	4.1 (2.9–5.3)	1.0 (0.5–2.2)
Black, non-Hispanic	15.0 (8.1–21.8)**	1.1 (0.6–2.0)	NA^††^	1.5 (0.5–4.5)
Hispanic	12.5 (8.0–17.1)	Ref	NA^††^	Ref
Other, non-Hispanic	17.2 (11.5–23.0)	1.4 (0.9–2.3)	NA^††^	1.6 (0.6–4.1)
**Education**				
High school diploma or less	10.2 (7.3–13.0)	Ref	4.9 (2.5–7.3)**	Ref
Some college	15.9 (11.6–20.2)	1.8 (1.2–2.5)	7.5 (3.5–11.5)**	1.8 (1.0–3.5)
College degree	15.7 (13.2–18.1)	2.2 (1.5–3.1)	3.5 (2.4–4.5)	1.2 (0.6–2.3)
**Employment status**				
Employed	15.6 (13.1–18.2)	1.5 (1.1–2.1)	6.1 (3.9–8.4)	2.1 (1.1–4.0)
Not employed	10.7 (8.0–13.3)	Ref	3.9 (1.9–5.8)**	Ref
**Marital status**				
Married	10.2 (8.0–12.5)	Ref	NA^††^	Ref
Not married	17.3 (14.3–20.3)	1.8 (1.2–2.5)	8.3 (5.6–10.9)	2.5 (1.1–6.0)
**Has a usual health care provider**				
Yes	11.9 (10.1–13.7)	Ref	4.4 (3.0–5.9)	Ref
No	17.8 (13.2–22.4)	1.7 (1.2–2.3)	7.2 (3.2–11.3)**	1.7 (0.8–3.3)
**Frequent mental distress** ^§§^				
Yes	27.4 (19.7–35.0)	2.3 (1.7–3.1)	15.3 (7.8–22.8)**	3.4 (1.9–5.8)
No	11.6 (9.8–13.3)	Ref	3.8 (2.4–5.1)	Ref

**Abbreviations:** aPR= adjusted prevalence ratio; NA = not available; Ref = referent group.

* Percentages weighted to represent national estimates of the U.S. population.

^†^ Defined as consuming at least one alcoholic drink in the past 30 days.

^§^ Defined as consuming four or more alcoholic drinks on one occasion at least once in the past 30 days.

^¶^ Model includes age, race/ethnicity, education, employment status, marital status, usual health care provider, and frequent mental distress.

** Estimate might be unstable because the relative SE is 0.2–0.3.

^††^ Estimate suppressed because the relative SE is >0.3.

^§§^ Defined as reporting ≥14 days of poor mental health in the past 30 days.

**FIGURE:**
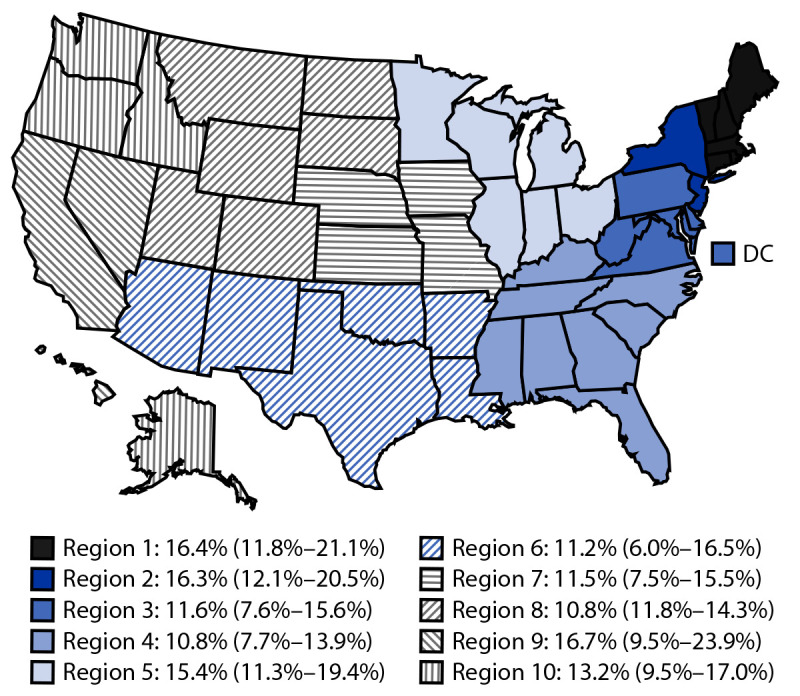
Estimated prevalence* of current drinking^†^ among pregnant adults aged 18–49 years (N = 6,327), by U.S. Department of Health and Human Services regions^§^ — Behavioral Risk Factor Surveillance System, United States, 2018–2020 **Abbreviation:** DC = District of Columbia. * Percentages weighted to represent national estimates of the U.S. population. Estimates for Region 9 and Region 6 might be unstable because the relative SEs are 0.2–0.3. ^†^ Defined as having consumed at least one alcoholic drink in the past 30 days. ^§^ *Region 1*: Connecticut, Maine, Massachusetts, New Hampshire, Rhode Island, and Vermont; *Region 2*: New Jersey and New York; *Region 3*: Delaware, District of Columbia, Maryland, Pennsylvania, Virginia, and West Virginia; *Region 4*: Alabama, Florida, Georgia, Kentucky, Mississippi, North Carolina, South Carolina, and Tennessee; *Region 5*: Illinois, Indiana, Michigan, Minnesota, Ohio, and Wisconsin; *Region 6*: Arkansas, Louisiana, New Mexico, Oklahoma, and Texas; *Region 7*: Iowa, Kansas, Missouri, and Nebraska; *Region 8*: Colorado, Montana, North Dakota, South Dakota, Utah, and Wyoming; *Region 9*: Arizona, California, Hawaii, and Nevada; *Region 10*: Alaska, Idaho, Oregon, and Washington.

## Discussion

During 2018–2020, approximately one in seven pregnant adults reported drinking alcohol in the past 30 days and, among those, approximately 40% reported binge drinking. Current and binge drinking increased by approximately 2 percentage points in 2018–2020 estimates compared with estimates from the previous 3-year period, consistent with an upward trend observed since 2011 (*2*,*4*). There was no evidence of increased alcohol consumption by pregnant adults in 2020 relative to 2019, despite possible increased alcohol sales and consumption among the general population during the first months of the COVID-19 pandemic (*5*).^§§§^

This report found several factors correlated with drinking during pregnancy including age, education, and marital status, which are generally consistent with other nationally representative studies (*4*,*6*). Having a usual health care provider was associated with lower alcohol consumption. Having a usual health care provider might increase receipt of prevention services including alcohol screening and brief intervention, an effective tool universally recommended in primary care settings (*7*,*8*). CDC is working to increase alcohol screening and brief intervention and community-level interventions.^¶¶¶^ Addressing barriers to having a usual health care provider might also help address alcohol consumption during pregnancy.

Frequent mental distress was correlated with current and binge drinking, although the direction of the relationship is unclear (*9*). Universal screening for anxiety and depression along with perinatal depression prevention interventions are recommended for women and pregnant adults.**** Integration of mental health services has been proposed in primary care setting and might be considered when addressing alcohol consumption during pregnancy (*3*).

The findings in this report are subject to at least five limitations. First, cross-sectional data limit inferences about temporal relationships. Second, low response rates could introduce selection bias. Third, data are self-reported and subject to misclassification related to recall and social desirability biases. Fourth, pregnancy might be misclassified because early pregnancies might be unrecognized. Finally, drinking was reported over a 30-day period which might not reflect drinking patterns earlier in pregnancy when consumption tends to be higher (*10*). These last three limitations might contribute to underestimates of drinking during pregnancy.

Alcohol consumption during pregnancy continues to be a serious problem. Addressing it requires clinical and community-wide interventions, such as alcohol screening and brief intervention and limiting alcohol sales. Improved access to care, including mental health services, might reduce prenatal alcohol use and prevent poor pregnancy and birth outcomes.

**Summary**

**What is already known about this topic?**

Alcohol consumption during pregnancy can cause fetal alcohol spectrum disorders and might increase the risk for poor pregnancy and birth outcomes. There is no known safe amount of alcohol consumption during pregnancy.

**What is added by this report?**

During 2018–2020, 13.5% of pregnant adults in the United States reported current drinking, and 5.2% reported binge drinking in the past 30 days. Those with no usual health care provider and those reporting frequent mental distress were more likely to consume alcohol.

**What are the implications for public health practice?**

High prevalence of alcohol consumption among pregnant adults requires integrated, evidence-based interventions to prevent alcohol-related harms and address factors associated with alcohol consumption.

## Footnotes

* New Jersey did not collect sufficient data to meet the minimum requirements for inclusion in 2019 BRFSS public use data set.

† Response rates were calculated using standards set by the American Association for Public Opinion Research. https://www.aapor.org/Standards-Ethics/Standard-Definitions-(1).aspx

§ Current drinking was defined as a response of “one or more” to the question, “During the past 30 days, how many days per week or per month did you have at least one drink of any alcoholic beverage such as beer, wine, a malt beverage or liquor?” https://www.cdc.gov/brfss/questionnaires/pdf-ques/2020-BRFSS-Questionnaire-508.pdf

¶ Binge drinking was defined as a response of “one or more” to the question, “Considering all types of alcoholic beverages, how many times during the past 30 days did you have four or more drinks on an occasion?”

** https://www.dietaryguidelines.gov/sites/default/files/2020-12/Dietary_Guidelines_for_Americans_2020-2025.pdf

†† Having a usual health care provider was ascertained by response to the question, “Do you have one person you think of as your personal doctor or health care provider?” Participants who answered “no” were asked, “Is there more than one, or is there no person who you think of as your personal doctor or health care provider?” Responses were dichotomized into one or more (yes) or none (no).

§§ Frequent mental distress was defined as a response of ≥14 to the question, “Now thinking about your mental health, which includes stress, depression, and problems with emotions, for how many days during the past 30 days was your mental health not good?” https://www.cdc.gov/hrqol/pdfs/mhd.pdf

¶¶ Sample sizes permitted examination of current drinking only by HHS region. https://www.hhs.gov/about/agencies/iea/regional-offices/index.html

*** 45 C.F.R. part 46, 21 C.F.R. part 56; 42 U.S.C. Sect. 241(d); 5 U.S.C. Sect. 552a; 44 U.S.C. Sect. 3501 et seq.

††† *Region 1*: Connecticut, Maine, Massachusetts, New Hampshire, Rhode Island, and Vermont; *Region 2*: New Jersey and New York; *Region 3*: Delaware, District of Columbia, Maryland, Pennsylvania, Virginia, and West Virginia; *Region 4*: Alabama, Florida, Georgia, Kentucky, Mississippi, North Carolina, South Carolina, and Tennessee; *Region 5*: Illinois, Indiana, Michigan, Minnesota, Ohio, and Wisconsin; *Region 6*: Arkansas, Louisiana, New Mexico, Oklahoma, and Texas; *Region 7*: Iowa, Kansas, Missouri, and Nebraska; *Region 8*: Colorado, Montana, North Dakota, South Dakota, Utah, and Wyoming; *Region 9*: Arizona, California, Hawaii, and Nevada; *Region 10*: Alaska, Idaho, Oregon, and Washington.

§§§ https://pubs.niaaa.nih.gov/publications/surveillance-covid-19/COVSALES.htm

¶¶¶ https://www.cdc.gov/ncbddd/fasd/alcohol-screening.html; https://www.thecommunityguide.org/topic/excessive-alcohol-consumption

**** The Well-Woman Chart outlines preventive services recommended by the Women’s Preventive Service Initiative, U.S. Preventive Services Task Force, and Bright Futures. https://www.womenspreventivehealth.org/wp-content/uploads/WPSI_WWC_11x17_2021Update.pdf
