# *Erratum:* Vol. 71, No. 1

**DOI:** 10.15585/mmwr.mm7104a4

**Published:** 2022-01-28

In the report “Alcohol Consumption and Binge Drinking During Pregnancy Among Adults Aged 18–49 Years — United States, 2018–2020,” in the figure on page 12, for Region 8, the values listed should have been 10.8% (**7.3%**–14.3%).

